# The role of dexamethasone during treatment phases in glioblastoma: Insights from a retrospective observational study

**DOI:** 10.1016/j.bas.2026.105968

**Published:** 2026-02-09

**Authors:** Juliane Göbel, Maximilian Scheer, Clemens Kirchner, Sandra Leisz, Julian Prell, Christian Strauss, Sebastian Simmermacher, Stefan Rampp

**Affiliations:** a Department of Neurosurgery, University Hospital Halle, Germany; b Department of Neurosurgery, Department of Neuroradiology, University Hospital Erlangen, Germany; c Department of Neurosurgery, University Hospital Heidelberg, Germany; d Department of Mathematics, ETH Zurich, Zurich, Switzerland

**Keywords:** Dexamethasone, Glioblastoma, Overall survival, Progression free survival, Cut-off

## Abstract

**Background:**

Glioblastoma (GBM) is the most aggressive primary brain tumor in adults. Dexamethasone (DEX) is commonly used to manage peritumoral edema, but its impact on overall survival (OS) and progression-free survival (PFS) remains unclear across treatment phases.

**Methods:**

In this retrospective single-center study, we analyzed data from 106 GBM patients treated between 2016 and 2020 at the University Hospital Halle. We examined the effects of DEX on OS and PFS during the preoperative, postoperative, and adjuvant therapy phases using Kaplan-Meier and Cox regression analyses. Cutoff analyses identified phase-specific DEX dose thresholds.

**Results:**

Preoperatively, DEX had no significant effect on OS (HR: 0.998, p = 0.379) or PFS (HR: 0.998, p = 0.373), though a positive trend is possible. Postoperatively, DEX was associated with improved OS (HR: 0.995, p = 0.017) and PFS (HR: 0.995, p = 0.029). Conversely, during adjuvant therapy, higher DEX doses trended toward worse OS (HR: 1.001, p = 0.069) and PFS (p = 0.258). Patients not receiving DEX during adjuvant therapy had significantly longer OS (17.9 vs. 6.4 months, p < 0.001) and PFS (9 vs. 4.6 months, p = 0.007).

**Conclusion:**

DEX influences survival outcomes differently across treatment phases. Higher doses may be beneficial pre- and postoperatively but detrimental during adjuvant therapy. These findings underscore the importance of phase-specific DEX dosing and support further research into optimal corticosteroid strategies in GBM care.

## Highlights

• Dexamethasone shows phase-dependent effects on survival in GBM patients, with potential benefits in the postoperative phase but negative trends during adjuvant therapy.
• Postoperative DEX use was associated with improved overall (HR: 0.995, p = 0.017) and progression-free survival (HR: 0.995, p = 0.029), while adjuvant phase use was linked to worse outcomes.
• Cutoff analyses identified critical DEX dose thresholds: >60 mg preoperatively, up to 140 mg postoperatively, and <40 mg during adjuvant therapy for favorable survival outcomes.
• Patients not receiving DEX during adjuvant therapy had significantly longer survival (OS: 17.9 vs. 6.4 months, p < 0.001; PFS: 9 vs. 4.6 months, p = 0.007), underscoring the need for cautious corticosteroid use.

## 1 Introduction

Glioblastoma (GBM) is the most common and aggressive primary brain tumor in adults, accounting for 14.5% of all central nervous system (CNS) tumors and 48.6% of malignant CNS tumors (Ostrom et al., 2019; Grochans et al., 2022). Although rare, it has an incidence of 3–4 cases per 100,000 people annually (Ostrom et al., 2019). Despite multimodal therapy, median survival remains limited at only 16 months [42].

Corticosteroids, particularly dexamethasone (DEX), are widely used in the management of GBM-associated peritumoral edema, which results from a disrupted blood-brain barrier (BBB). DEX reduces edema by modulating the expression of tight junction proteins such as claudin-5, occludin, and ZO-1, thereby restoring BBB integrity (Blecharz et al., 2010; Hue et al., 2015; Romero et al., 2003). The anti-edema effects of DEX are rapid, with symptom relief observed within 24–48 h (Shapiro et al., 1990; Drappatz et al., 2007). This makes it the treatment of choice for managing intracranial pressure (ICP) and neurological symptoms in GBM patients (Galicich et al., 1961; Jelsma and Bucy, 1967).

Despite its benefits, the use of DEX in GBM therapy is controversial due to its broad immunosuppressive effects and potential adverse outcomes. Studies suggest that DEX may impair the efficacy of adjuvant tumor treatments and immunotherapies, potentially leading to worse overall survival (OS) and progression-free survival (PFS) outcomes (Shields et al., 2015; Wong et al., 2015; Iorgulescu et al., 2021). Additionally, DEX may promote glioblastoma cell proliferation, invasion, and angiogenesis (Luedi et al. 2017, 2018). Given its wide-ranging effects, understanding the impact of DEX on survival outcomes across different treatment phases is of critical importance.

This retrospective, single-center study evaluates the influence of DEX administration on OS and PFS in glioblastoma patients throughout various treatment phases. By analyzing data from 106 patients, we explore potential associations between DEX dosage, timing, and survival outcomes, contributing to the ongoing debate on the optimal use of corticosteroids in GBM management.

## 2 Methods

### 2.1 Study design and population

**Fig. 1:**
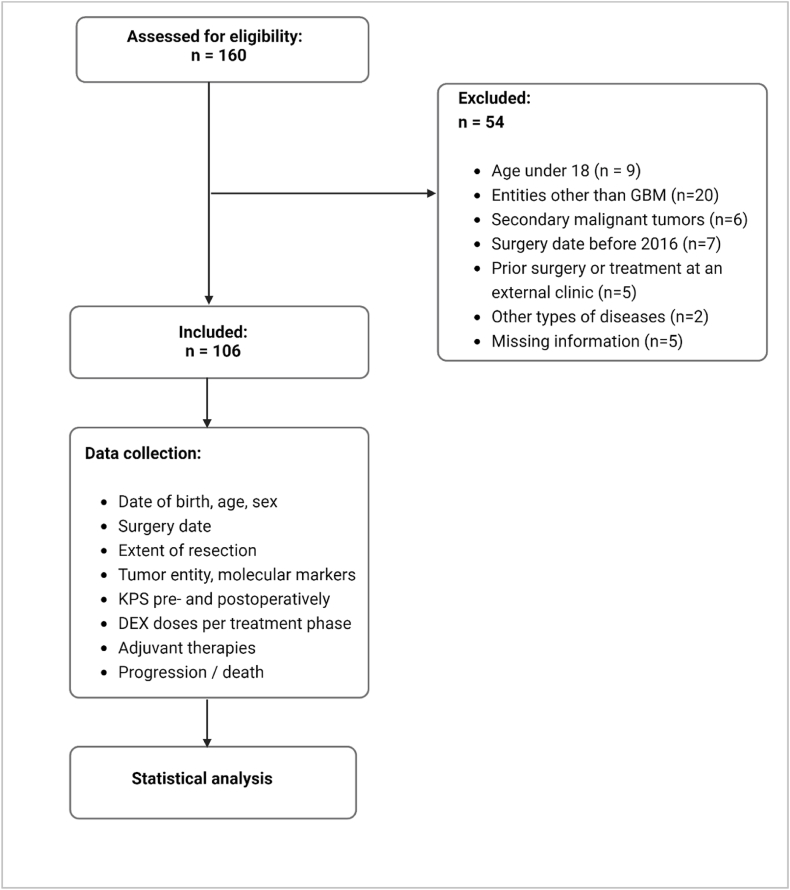
Flow chart of the study.

This retrospective, single-center study initially screened 160 patients with newly diagnosed GBM treated at the University Hospital Halle (Saale), Germany, between 2016 and 2020. Of these, 106 patients were deemed eligible for inclusion. Patients were included if they had histologically confirmed GBM WHO grade 4 based on the 2016 WHO classification (Louis et al., 2016) and complete clinical data. Exclusion criteria were age under 18, previous surgery before 2016, non-GBM pathologies, or incomplete data on DEX use, progression-, or survival. Patient selection and the overall study framework are outlined in the flow chart (Fig. 1). The study protocol was approved by the Ethics Committee of the Medical Faculty of the Martin Luther University Halle-Wittenberg prior to study initiation (processing number 2021-192).

### 2.2 Data collection

Clinical and treatment data were collected from the hospital's electronic medical records. Key variables included demographics, extent of resection (biopsy, STR (subtotal-), or GTR (gross-total resection)), MGMT promoter methylation, IDH mutation status, pre- and postoperative Karnofsky Performance Status (KPS), adjuvant therapies, and cumulative DEX doses during preoperative, postoperative, and adjuvant phases. Survival outcomes were assessed up to December 31, 2022.

### 2.3 Outcome measures

The primary endpoints were OS and PFS. OS was defined as the time from surgery to death from any cause, while PFS was defined as the time from surgery to progression or death. Progression was assessed according to the Response Assessment in Neuro-Oncology (RANO) criteria, which standardize the evaluation of tumor burden and clinical status (Wen et al., 2010).

### 2.4 Statistical analysis

As this is an exploratory study, no predefined significance level or sample size calculation was performed. P-values were interpreted in the exploratory context. Descriptive statistics were calculated for the collected data. For categorical variables, absolute and relative frequencies were reported, while for continuous variables, mean, standard deviation (SD), median, 1st and 3rd quartiles, interquartile range (IQR), and range (minimum and maximum) were provided. Continuous and categorical variables were reported as medians with an interquartile range and proportions. Between-group differences were tested using Wilcoxon rank-sum tests for continuous variables and chi-square tests for categorical variables. For comparisons across more than two groups, the Kruskal-Wallis test was applied to assess differences in continuous variables. To assess linear relationships between two continuous variables, Spearman's rank correlation test was applied.

Survival statistics were calculated using the Kaplan-Meier method, and differences were evaluated with log-rank tests. Cox proportional hazards models were used to assess the association of DEX doses with OS and PFS, adjusted for key clinical variables. To assess the impact of the variables, hazard ratio (HR) with 95% confidence intervals (CI) were calculated. Hazard ratios (HR) with 95% confidence intervals (CI) were calculated to evaluate the impact of these variables. Cut-off analyses were performed to identify thresholds for cumulative DEX doses associated with survival differences, with these thresholds being determined in an exploratory manner. All analyses, as well as their graphical and tabular representation, were performed using the R programming language, version 4.2.2 (R Core Team, Vienna, Austria; Developers: Robert Gentleman and Ross Ihaka, University of Auckland, New Zealand). Individual tables were created using Word. A flow chart of the study is shown in Fig. 1.

## 3 Results

### 3.1 Study population

The study cohort consisted of 106 patients with newly diagnosed GBM. Baseline characteristics are summarized in Table 1. The cohort included 61 male (57.5 %) and 45 female (42.5 %) patients, with a mean age of 66 years (range: 38–87 years) at diagnosis. Tumor localization was predominantly supratentorial (86 %), with infratentorial (8.4 %), multilocal (5.6 %) cases also represented.

**Table 1:** Baseline features of the study population.

Patient Characteristic	Value
**Total patients (N)**	106
**Age at diagnosis, years**	
Median (IQR)	66,0 (57.2 – 75.0)
Mean (SD)	66,3 (10,6)
Range	38 - 87
**Gender, n/N (%)**	
Male	61/106 (57.5%)
Female	45/106 (42.5%)
**Extent of resection, n/N (%)**	
Biopsy	37/106 (34.9%)
GTR	48/106 (45.3%)
STR	21/106 (19.8%)
**MGMT promoter methylation, n/N (%)**	
Yes	55/106 (51.9%)
No	39/106 (36.8%)
Not determined	12/106 (11.3%)
**IDH1/2-Mutation, n/N (%)**	
Yes	1/106 (1%)
No	95/106 (89.6%)
Not determined	10/106 (9.4%)
**Preoperative KPS**	
Median (IQR)	70 (60 - 80)
Mean (SD)	68,5 (14,3)
Range	40 – 90
**Postoperative KPS**	
Median (IQR)	70 (50 - 80)
Mean (SD)	64 (18,6)
Range	0 – 90
**Adjuvant therapies, n/N (%)**	
RCT + chemotherapy	51/106 (48.1%)
RCT only	17/106 (16%)
Radiotherapy only	20/106 (18.9%)
None	18/106 (17%)
**TTFields, n/N (%)**	
Yes	20 (19%)
No	86 (81%)

GTR was achieved in 48 patients (45.3 %), while 21 (19.8 %) underwent STR, and 37 (34.9 %) had only a biopsy. Molecular analysis showed MGMT promoter methylation in 55 cases (51.9 %), while IDH mutations were rare (1 %). Preoperative Karnofsky Performance Status (KPS) had a median of 70 (range: 40–90), which remained unchanged postoperatively (median: 70, range: 0–90).

Adjuvant therapy was administered in 88 patients (83 %), with 51 (48.1 %) receiving concurrent radiotherapy and chemotherapy (RCT), followed by adjuvant chemotherapy. In 17 patients (16 %), only RCT was conducted, and tumor-treating fields (TTFields) were used in 20 cases. Hypofractionated or palliative radiotherapy was performed in 20 patients (18.9 %). Baseline features were summarized in Table 1.

### 3.2 DEX administration

All patients received DEX during their treatment, with doses ranging from 1 mg to 44 mg per day, depending on symptoms and treatment phase. The dosage varied between patients and was not consistent over time. DEX was administered to all patients in both the preoperative and postoperative phases, while 56 out of 88 (64%) patients received DEX during the adjuvant therapy phase.

The median cumulative dose of DEX in the preoperative phase was 107 mg (range: 20–275 mg), with a median duration of 7 days (range: 2–34 days). In the postoperative phase, the median cumulative dose was 134 mg (range: 82–350 mg), and the median duration was 21 days (range: 7–62 days). During the adjuvant therapy phase, the median cumulative dose of DEX was 30.75 mg (range: 0–712.5 mg), with a median duration of 40 days (range: 6–96 days). Among the 67 patients who received RCT, 41 patients were prescribed DEX, with a median dose of 20.5 mg (range: 0–596 mg). For the 21 patients who received radiotherapy alone, the median dose was 56 mg (range: 0–712.5 mg).

**Fig. 2:**
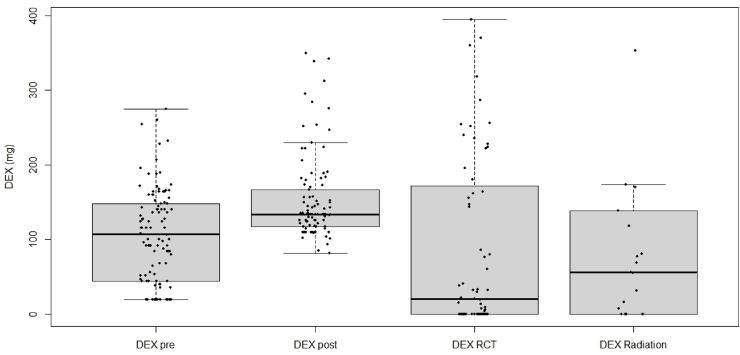
Box plots for the distribution of the cumulative DEX dose during the different treatment phases.

The total cumulative DEX dose, combining all phases of administration, was 304 mg in the median (range: 130–1018 mg). The cumulative doses are visualized as box plots in Fig. 2.

### 3.3 General survival outcomes

A total of 106 patients were included; 101 had died by the end of follow-up and 5 were alive at last follow-up. Median OS was 229 days (7.5 months; 95% CI 5.8–10.0) and median PFS was 137 days (4.5 months; 95% CI 4.0–5.5), with OS ranging from 6 to 1700 days and PFS from 6 to 1401 days. Survival differed by extent of resection, with longest outcomes after GTR (median OS 12.3 months; PFS 6.4 months), followed by STR (OS 8.4; PFS 4.2), and biopsy (OS 3.5; PFS 3.2). Adjuvant therapy was strongly associated with improved survival: radiochemotherapy yielded median OS/PFS of 15.1/7.3 months, increasing to 18.2/9.4 months with additional tumor-treating fields. Radiotherapy alone resulted in OS/PFS of 4.8/4.2 months, while no adjuvant therapy showed the poorest outcomes (OS and PFS 1.5 months). Kaplan–Meier curves are shown in Supplementary Figs. S1 and S2.

### 3.4 Analysis of DEX effects on preoperative and postoperative survival outcomes

The multivariate Cox regression analyses for OS and PFS in the preoperative and postoperative phases, specifically focusing on the effects of DEX, are summarized in Tables 2.

**Table 2:** Cox regression for OS and PFS pre- and postoperatively.

Variable	HR^a^	95% CI^a^	p-value
**DEX pre per mg**	0.998	0.995, 1.002	0.379
**DEX post per mg**	0.995	0991, 0.999	0.017
**Extent of resection**			
**Biopsy**	-	-	
**GTR**	0.447	0.266, 0.751	0.002
**STR**	0.626	0.340, 1.152	0.132
**KPS prä**	0.998	0.978, 1.018	0.811
**KPS post**	0.962	0.941, 0.984	<0.001
**Adjuvant therapy**			
**None**	-	-	
**Radiation**	0.155	0.062, 0.384	<0.001
**RCT**	0.049	0.019, 0.130	<0.001

Variable	HR^a^	95% CI^a^	p-value
**DEX pre per mg**	0.998	0.995, 1.002	0.373
**DEX post per mg**	0.995	0991, 1.000	0.029
**Extent of resection**			
Biopsy	-	-	
GTR	0.506	0.305, 0.840	0.008
STR	0.530	0.282, 0.993	0.047
**KPS prä**	1.005	0.986, 1.025	0.583
**KPS post**	0.969	0.948, 0.989	0.003
**Adjuvant therapy**			
None	-	-	
Radiation	0.156	0.069, 0.350	<0.001
RCT	0.087	0.038, 0.202	<0.001

a HR = Hazard Ratio, CI = Confidence Interval.

In the preoperative phase, DEX administration showed no clear impact on both OS (HR = 0.998; 95% CI [0.995–1.002]; p = 0.379) and PFS (HR = 0.998; 95% CI [0.995–1.002]; p = 0.373), but a potential positive trend cannot be excluded.

In the postoperative administration of DEX showed a positive effect on both OS (HR = 0.995; 95% CI [0.991–0.999]; p = 0.017) and PFS (HR = 0.995; 95% CI [0.991–1.000]; p = 0.029). For every additional 1 mg administered postoperatively, OS was extended by approximately 0.5%.

### 3.5 Analysis of DEX effects on adjuvant therapy survival outcomes

The multivariate Cox regression analysis for OS and PFS during the adjuvant therapy phase was conducted in 88 patients who received adjuvant therapy (Table 3). The results indicated a trend toward a negative impact on OS (HR = 1.001; 95% CI [1.000–1.003]; p = 0.069) and PFS (HR = 1.001; 95% CI [0.999–1.002]; p = 0.258).

**Table 3:** Cox regression for PFS and OS during adjuvant treatment.

Variable	HR^a^	95% CI^a^	p-value
**DEX adj per mg**	1.001	1.000, 1.003	0.069
**Extent of resection**			
Biopsy	-	-	
GTR	0.593	0.343, 1.023	0.061
STR	0.789	0.401, 1.553	0.492
**KPS post**	0.972	0.950, 0.994	0.012
**Adjuvant therapy**			
Radiation	-	-	
RCT	0.293	0.157, 0.547	<0.001

Variable	HR^a^	95% CI^a^	p-value
**DEX adj per mg**	1.001	0.999, 1.002	0.258
**Extent of resection**			
Biopsy	-	-	
GTR	0.613	0.356, 1.055	0.077
STR	0.805	0.407, 1.590	0.531
**KPS post**	0.990	0.969, 1.011	0.353
**Adjuvant therapy**			
Radiation	-	-	
RCT	0.461	0.257, 0.824	0.009

a HR = Hazard Ratio, CI = Confidence Interval.

Furthermore, a group comparison was performed for the adjuvant therapy phase, which included both patients who received DEX and those who did not. The results revealed that patients who did not receive DEX during adjuvant therapy had longer OS (median: 17.9 months vs. 6.4 months, p < 0.001) and PFS (median: 9 months vs. 4.6 months, p = 0.007) compared to those who were treated with DEX. No notable differences were observed between the groups regarding age, gender, preoperative KPS, postoperative KPS, and extent of tumor resection in the preoperative and postoperative phases (Table 4).

**Table 4:** Group comparison DEX administration during adjuvant treatment.

Variable	DEX, n = 56^a^ (95% CI)	No DEX, n = 32^a^ (95% CI)	p-value b
**Age**			0.2
Mean	66.2 (63, 69)	63.3 (60, 67)	
**Gender**			0.2
male	29/56 (52%) (38%, 65%)	21/32 (66%) (47%, 81%)	
female	27/56 (48%) (35%, 62%)	11/32 (34%) (19%, 53%)	
**KPS pre**			0.8
Mean	70.4 (67, 74)	71.3 (66, 76)	
**KPS post**			0.8
Mean	68.9 (65, 72)	70.6 (67, 75)	
**Extent of resection**			0.2
Biopsy	19/56 (34%) (22%, 48%)	5/32 (16%) (5.9%, 34%)	
GTR	25/56 (45%) (32%, 58%)	20/32 (63%) (44%, 78%)	
STR	12/56 (21%) (12%, 35%)	7/32 (22%) (9.9%, 40%)	
**OS**			<0.001
Mean	349.7 (257, 424)	610.4 (471, 749)	
Median	194.0 (164, 293)	543.0 (441, 722)	
**PFS**			0.007
Mean	216.6 (159, 274)	338.2 (238, 439)	
Median	140.5 (122, 168)	274.0 (159, 414)	

a Group size.

b Wilcoxon rank sum test; Pearson's Chi-squared test.

### 3.6 Threshold values for DEX administration

In the Cox regression analyses, preoperative and postoperative DEX administration suggested a potential positive association with OS, whereas DEX during the adjuvant phase appeared to be associated with lower survival. Cutoff analyses were conducted to investigate threshold values for cumulative DEX dosage in the respective phases, with these thresholds identified through an exploratory approach.

**Fig. 3:**
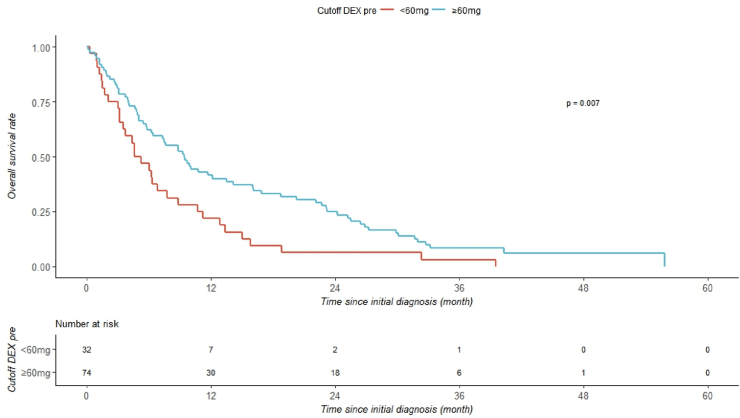
Kaplan-Meier curve of the preoperative phase for patients with a DEX dose of more than or less than 60 mg.

For the preoperative phase, a lower threshold of 60 mg was identified. Patients who received less than 60 mg showed a shorter median OS compared to those with at least 60 mg (4.9 months vs. 9.4 months, p = 0.007; log-rank test, Fig. 3).

**Fig. 4:**
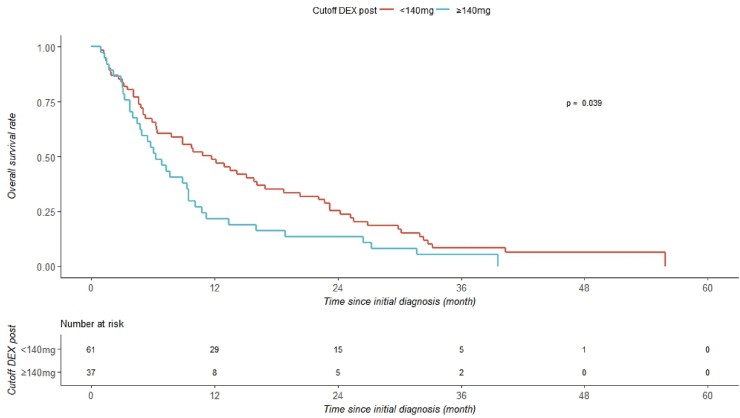
Kaplan-Meier curve of the postoperative phase for patients with a DEX dose of more than or less than 140 mg.

In the postoperative phase, an upper threshold of 140 mg was identified. Patients who received ≥140 mg showed a shorter median OS compared to those who received lower doses (6.3 months vs. 10.8 months, p = 0.039; log-rank test, Fig. 4). In combination with the results from the Cox regression analysis, these findings suggest that postoperative DEX may be associated with a beneficial effect on survival, but only up to an threshold of 140 mg.

**Fig. 5:**
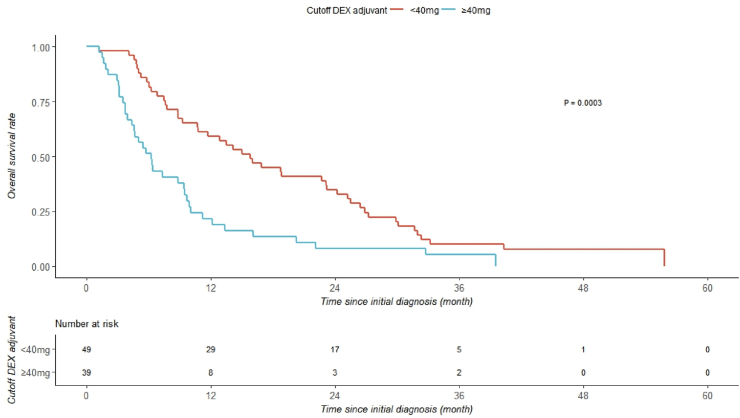
Kaplan-Meier curve during adjuvant treatment for patients with a DEX dose of more than or less than 40 mg.

In the adjuvant therapy phase, a threshold of 40 mg was identified (5.8 months vs. 15.9 months, p = 0.0003; log-rank test, Fig. 5). Patients who received ≥40 mg showed a shorter median overall survival compared to those who received less.

### 3.7 DEX dosage and patient characteristics

**Fig. 6:**
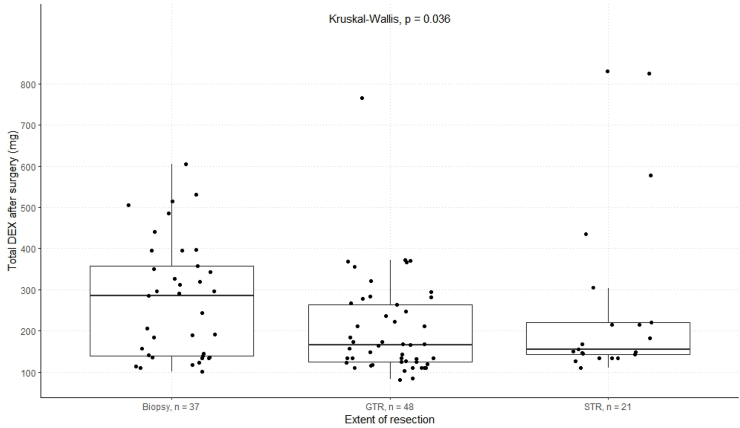
Box plots for the distribution of the cumulative DEX dose depending on the extent of resection.

DEX dosage varied by patient characteristics, including surgical procedure, age, and functional status. During therapy, the biopsy group received the highest median DEX dose (284.5 mg), higher than in patients who received GTR or STR (165.5 mg and 154.5 mg, respectively, p = 0.036, Fig. 6).

**Fig. 7:**
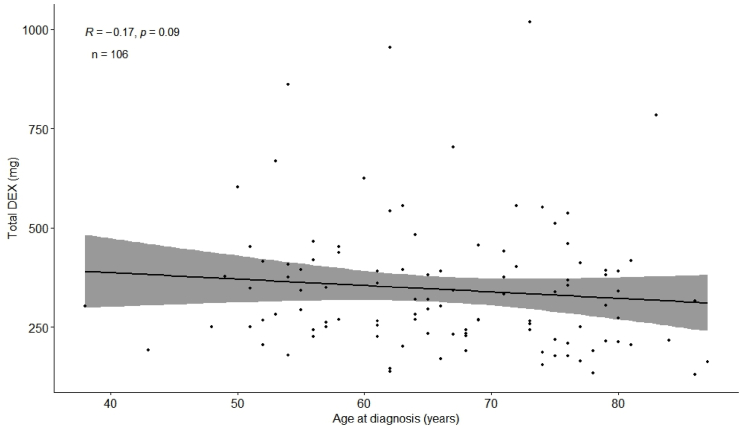
Scatterplot shows correlation of cumulative DEX dosage and age.

A weak negative correlation was observed between patient age and cumulative DEX dosage (R = −0.17, p = 0.09, Fig. 7). However, differences were not notable, and the scatterplot does not indicate a clear relationship.

**Fig. 8:**
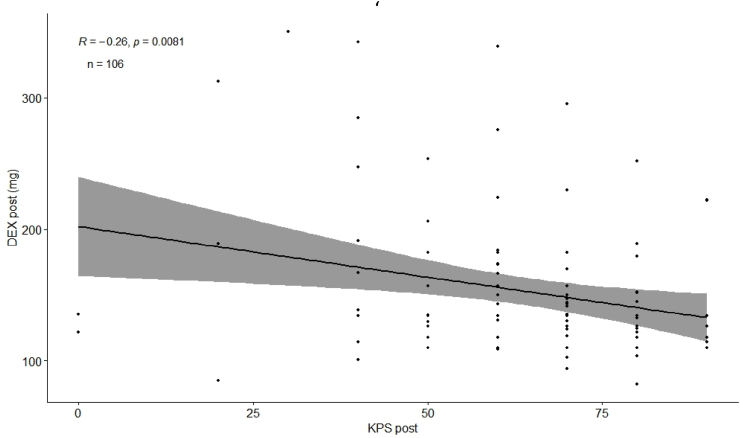
Scatterplot shows correlation of postoperative DEX and postoperative KPS.

Postoperative functional status (KPS) was negatively correlated with postoperative DEX dosage (R = −0.23, p = 0.018, Fig. 8), indicating that lower KPS values were associated with higher DEX doses. A 10-point decrease in KPS corresponded to an increase of 7 mg in postoperative DEX dosage.

## 4 Discussion

DEX remains the standard of care to control perifocal edema in brain tumor patients and is routinely administered perioperatively to prevent malignant brain swelling (Afshari et al., 2022). However, DEX dosing varies widely between institutions, as many centers rely on internal, experience-based regimens due to limited evidence on the optimal dose and schedule (Lim-Fat et al., 2019; Jessurun et al., 2019). Unfortunately, there are no prospective randomised trials that examine the dosage in detail. Many studies on which current treatments are based are outdated (Jessurun et al., 2019).

Clinically, DEX effectively reduces edema-related symptoms, lowering intracranial pressure, improving neurological deficits, and increasing functional status (KPS) (Alberti et al., 1978; Palombi et al., 2018; Villani et al., 2019). At the same time, higher perioperative dosing is linked to relevant toxicity, including increased delirium risk and infectious complications, likely driven by its immunosuppressive effects (Flanigan et al., 2018; Koning et al., 2021; Wu et al., 2021; Mistry et al., 2021). In GBM patients, DEX has been shown to markedly reduce circulating immune cell populations (B, T, and NK cells) (Chitadze et al., 2017; Bracci et al., 2022). Importantly, postoperative doses >8 mg/day were associated with hyperglycemia and higher complication rates, including thrombosis, bleeding, and infections (Jatana et al., 2023).

Several studies and recent reviews indicate that DEX exposure—particularly during adjuvant therapy—may negatively affect PFS and OS in GBM (Wong et al., 2015; Pitter et al., 2016; Petrelli et al., 2021; Scheffler et al., 2024; Arora et al., 2024). Wong et al. reported significantly shorter survival in patients receiving >4.1 mg/day, and Pitter et al. identified DEX during radiotherapy as an independent risk factor for reduced OS (Wong et al., 2015; Pitter et al., 2016). Mechanistically, DEX may reduce sensitivity to radio-/chemotherapy by promoting tumor cell survival, inhibiting apoptosis, and potentially inducing MGMT-mediated TMZ resistance (Aasland et al., 2018; Aldaz et al., 2021; Komakech et al., 2020).

Across preclinical and clinical evidence, dexamethasone consistently emerges as a critical negative modulator of immunotherapy efficacy in glioma. In multiple cellular and murine glioma models, concurrent dexamethasone administration dose-dependently reduced survival benefits of anti-PD-1 therapy and could even abrogate responses to checkpoint blockade with or without radiotherapy, particularly in immune-resistant settings. Mechanistically, dexamethasone reduced key immune effector populations by promoting T-cell apoptosis and impairing lymphocyte function, while also diminishing myeloid and natural killer cell compartments, indicating suppression of both adaptive and innate immunity (Iorgulescu et al., 2021). A broader synthesis of 21 preclinical studies and 3 clinical studies further supports that dexamethasone decreases microglia/macrophage presence and T-lymphocyte numbers in tumor tissue and periphery, and blunts checkpoint inhibitor–mediated antitumor activity (Swildens et al., 2022). Complementary functional data demonstrate that while dexamethasone only slightly affects direct gene-mediated cytotoxic immunotherapy (GMCI) cytotoxicity in vitro, it markedly compromises T-cell killing capacity and proliferation, translating in vivo into significantly reduced median symptom-free survival when combined with GMCI (29 vs. 39.5 days; P = 0.0184) (Koch et al., 2019). Clinically, although randomized data are lacking, retrospective analyses in GBM patients receiving PD-(L)1 blockade indicate worse survival outcomes in those treated with baseline corticosteroids, even after adjustment for relevant prognostic factors (Iorgulescu et al., 2021).

In our cohort, DEX was analyzed separately for preoperative, postoperative, and adjuvant phases, enabling dose-specific cut-off definitions. We identified 60 mg preoperatively, 140 mg postoperatively, and 40 mg during adjuvant therapy as relevant thresholds. While DEX showed an overall negative association with outcome—consistent with the literature—postoperative DEX up to 140 mg was associated with improved PFS and OS, a finding not previously reported and potentially attributable to our more granular phase-specific categorization. Notably, patients with higher postoperative KPS received less DEX, and biopsy-only patients received higher cumulative doses overall.

Given the potential harm of prolonged or higher-dose DEX—especially during adjuvant therapy—several centers increasingly aim for restrictive use and explore alternatives (e.g., bevacizumab or RAGE-pathway inhibition). A prospective trial at Inselspital Bern is currently evaluating perioperative outcomes without DEX (NCT04266977.

## 5 Limitations

This retrospective, single-center study has inherent limitations that may affect the generalizability of the findings. Selection bias cannot be excluded, as treatment decisions regarding DEX dosing were made on an individual basis rather than following a standardized protocol. Additionally, data collection relied on electronic records, posing a risk of information bias, particularly regarding the accuracy of recorded DEX dosages and potential discrepancies in outpatient adherence.

While we analyzed cumulative DEX doses within distinct treatment phases, variations in individual tapering regimens and potential post-adjuvant DEX use were not accounted for, which may influence survival outcomes. Furthermore, clinical deterioration or other factors influencing DEX prescription were not systematically documented, limiting insight into the underlying reasons for dose adjustments.

Given these limitations, prospective studies with standardized DEX protocols and comprehensive longitudinal assessments are needed to validate these findings and further clarify the optimal corticosteroid management in GBM.

## 6 Conclusion

In general, the use of DEX in GBM patients should be carefully reconsidered. While DEX can be used for symptom control in GBM patients, its potential impact on survival varies across treatment phases. In the adjuvant setting, DEX should be avoided if possible. However, in the immediate postoperative period, our findings suggest that DEX may be beneficial on OS and PFS up to a cumulative dose of 140 mg. The principle of maximal safe resection remains important to achieve the best possible KPS and to allow adjuvant therapy. Future prospective studies are needed to define optimal DEX thresholds that balance symptom control with potential treatment-related risks.

## Contributions

Conceptualization, M.S. and S.S.; methodology, J.G. and M.S.; formal analysis, J.G., S.R. and C.K.; resources, J.*P. and* C.S.; data curation, J.G. and M.S.; writing—original draft preparation, J.G. & M.S.; writing—review and editing, S.S., S.R. and S.L.; visualization, C.K. and J.G.; supervision, S.S. and S.R.; project administration, M.S. and S.S.; funding acquisition, M.S. All authors have read and agreed to the published version of the manuscript for the term explanation.

## Human ethics and consent to participate declarations

Not applicable.

## Ethics approval

The study protocol was approved by the Ethics Committee of the Medical Faculty of the Martin Luther University Halle-Wittenberg prior to study initiation (processing number 2021-192).

## Declaration of competing interest

The authors declare that they have no known competing financial interests or personal relationships that could have appeared to influence the work reported in this paper.
