# Supplementary material for: The role of dexamethasone during treatment phases in glioblastoma: Insights from a retrospective observational study
Source: Brain Spine. 2026 Feb 9;6:105968. doi: 10.1016/j.bas.2026.105968 (PMC12925272; doi:10.1016/j.bas.2026.105968)
Supplement: Multimedia component 1 [file mmc1.docx]

Supplementary Figures:


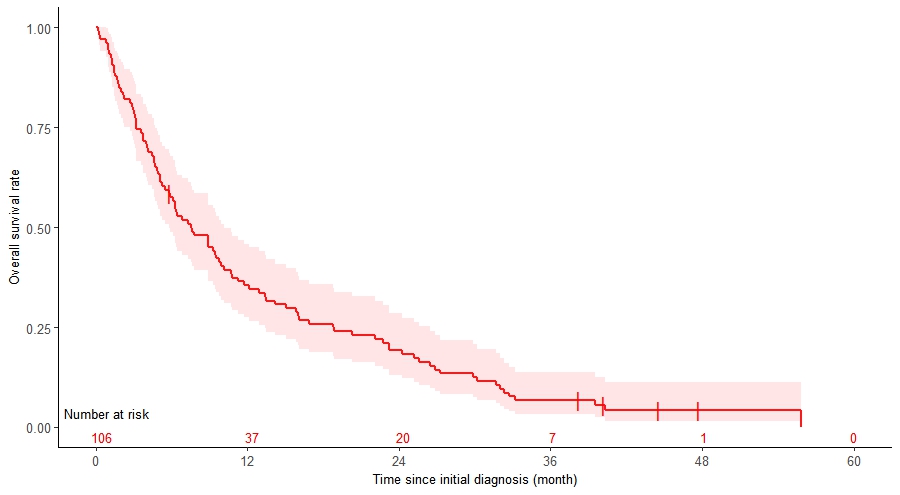


**Figure S1:** Kaplan-Meier curve for OS of our study cohort. Median OS was 7.5 months.


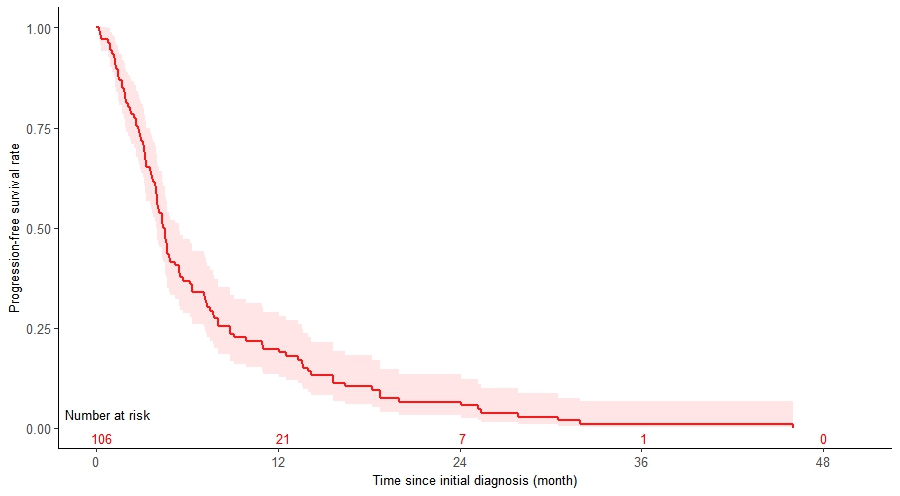


**Figure S2:** Kaplan-Meier curve for PFS of our study cohort. Median PFS was 4.5 months.
